# A Comparative Analysis of CNN Architectures, Fusion Strategies, and Explainable AI for Fine-Grained Macrofungi Classification

**DOI:** 10.3390/biology14121733

**Published:** 2025-12-03

**Authors:** Mustafa Sevindik, Aras Fahrettin Korkmaz, Fatih Ekinci, Eda Kumru, Ömer Burak Altındal, Alperen Aydın, Mehmet Serdar Güzel, Ilgaz Akata

**Affiliations:** 1 Department of Biology, Faculty of Engineering and Natural Sciences, Osmaniye Korkut Ata University, Osmaniye 80000, Türkiye; 2 Nutrition and Dietetics Department, Faculty of Health Sciences, Şirinevler Campus, İstanbul Kültür University, Istanbul 34191, Türkiye; a.korkmaz@iku.edu.tr; 3 Institute of Artificial Intelligence, Ankara University, Ankara 06100, Türkiye; fatihekinci@ankara.edu.tr; 4 Graduate School of Natural and Applied Sciences, Ankara University, Ankara 06830, Türkiye; ekumru@ankara.edu.tr; 5 Department of Computer Engineering, Faculty of Engineering, Ankara University, Ankara 06830, Türkiye; omeraltindal6800@gmail.com (Ö.B.A.); mguzel@ankara.edu.tr (M.S.G.); 6 Artificial Intelligence and Data Engineering, Faculty of Engineering, Ankara University, Ankara 06830, Türkiye; 22290435@ogrenci.ankara.edu.tr; 7 Department of Biology, Faculty of Science, Ankara University, Ankara 06100, Türkiye

**Keywords:** macrofungi classification, deep learning, convolutional neural networks, model fusion, explainable AI, coprinoid mushrooms

## Abstract

This study was motivated by the persistent difficulty of accurately identifying morphologically similar macrofungi species, which remains a significant challenge in fungal taxonomy and biodiversity monitoring. This study presents a deep learning framework for the automated classification of seven morphologically similar coprinoid macrofungi species. A curated dataset of 1692 high-resolution images was used to evaluate ten state-of-the-art convolutional neural networks (CNNs) and three novel fusion models. The Dual Path Network (DPN) achieved the highest performance as a single model with 89.35% accuracy, a 0.8764 Matthews Correlation Coefficient (MCC), and a 0.9886 Area Under the Curve (AUC). The feature-level fusion of Xception and DPN yielded competitive results, reaching 88.89% accuracy and 0.8803 MCC, demonstrating the synergistic potential of combining architectures. In contrast, lighter models like LCNet and MixNet showed lower performance, achieving only 72.05% accuracy. Explainable AI (XAI) techniques, including Grad-CAM and Integrated Gradients, confirmed that high-performing models focused accurately on discriminative morphological structures such as caps and gills. The results underscore the efficacy of deep learning, particularly deeper architectures and strategic fusion models, in overcoming the challenges of fine-grained visual classification in mycology. This work provides a robust, interpretable computational tool for automated fungal identification, with significant implications for biodiversity research and taxonomic studies.

## Simple Summary

This research developed an artificial intelligence system to automatically identify seven similar-looking mushroom species from the coprinoid group. We used a collection of 1692 mushroom photos to test different AI models. The best-performing single model, called DPN, correctly identified mushrooms 89.35% of the time. Combining two models (Xception and DPN) also worked very well, achieving 88.89% accuracy. Simpler, lighter models were less accurate, with the lowest scoring only 72.05%. The AI system was designed to be transparent, showing that it makes decisions by looking at key mushroom parts like caps and stems, much like a human expert would. This technology offers a powerful new tool to help mycologists and nature enthusiasts quickly and accurately identify these fungi in the field.

## 1. Introduction

Coprinoid fungi, often referred to as “inky caps,” are a distinctive group within the *Agaricales* (*Basidiomycota*), recognized for their unique shape and ecological significance. They exhibit deliquescence, meaning their gills autodigest and turn into inky liquid to disperse spores [1]. These fungi typically grow on decaying wood, dung, compost, leaf litter, and nutrient-rich soils [2]. Traditionally, all coprinoid species were classified under the genus *Coprinus* Pers. due to their shared deliquescent appearance [3,4]. However, molecular studies have shown that this group is polyphyletic, leading to its reclassification. Currently, *Coprinus* sensu stricto includes only *Coprinus comatus* (O.F. Müll.) Pers. and related species within the family *Agaricaceae* Chevall., while other species have been moved to *Coprinellus* P. Karst., *Coprinopsis* P. Karst., and Parasola Redhead, Vilgalys & Hopple in the family *Psathyrellaceae* Vilgalys, Moncalvo & Redhead [1,2,3,4,5,6]. Distinguishing coprinoid macrofungi species is inherently challenging due to their highly similar morphologies, overlapping habitats, and rapid developmental changes. Manual identification requires considerable taxonomic expertise, is time-consuming, and may result in inconsistencies among experts. These limitations highlight the need for automated, reliable, and high-accuracy computational systems capable of supporting experts in differentiating these closely related species.

Various species showcase the ecological and biological diversity of coprinoid fungi. *Coprinellus disseminatus* (Pers.) J.E. Lange is a widespread species often found in dense clusters at the bases of stumps, producing thousands of small, non-deliquescent fruiting bodies [7]. *Coprinellus micaceus* (Bull.) Vilgalys, Hopple & Jacq. Johnson, a standard ink cap, frequently grows in large groups on buried wood and is easily identified by its shimmering, mica-like veil remnants [8]. *Coprinellus domesticus* (Bolton) Vilgalys, Hopple & Jacq. Johnson, often associated with decaying wood, features an orange-brown mycelial mat known as ozonium [9].

The genus *Coprinopsis* includes several species of ecological and chemical significance [10]. The most well-known is *Coprinopsis atramentaria*, recognized for its widespread presence and for containing coprine, which causes disulfiram-like reactions with alcohol, highlighting its toxicity [11]. Other notable species include *Coprinopsis lagopus*, a delicate species with a fast life cycle, and *Coprinopsis picacea*, known for its striking cap pattern that resembles magpie plumage [9]. Finally, *Coprinus comatus*, known as the shaggy ink cap, is a recognizable coprinoid fungus valued for its culinary and nutritional benefits [5]. Its tall, shaggy stem and delayed deliquescence differentiate it morphologically from other ink caps, supporting its classification within Coprinus sensu stricto in the family *Agaricaceae* [1].

A deep learning-based approach was adopted for the automatic classification of macrofungi species, systematically evaluating ten state-of-the-art convolutional neural network (CNN) architectures—EdgeNeXT [12], RepVGG [13], DPN [14], GhostNet [15], Xception [16], EfficientNetB0 [17], EfficientNetB4 [18], LCNet [19], MixNet [20], and MobileNetV2 [21]—and three custom fusion models: RepVGG + EdgeNeXT, MixNet + EfficientNetB4, and Xception + DPN [22,23,24]. To enhance model performance and improve generalization, a comprehensive preprocessing and data augmentation pipeline was applied to the dataset [25]. Furthermore, Explainable Artificial Intelligence (XAI) techniques, such as Gradient-weighted Class Activation Mapping (Grad-CAM) and Integrated Gradients, were employed to ensure the transparency of the models’ decision-making processes and to verify their focus on biologically meaningful features [26,27]. This comprehensive approach enabled the evaluation of not only classification accuracy but also the reliability and interpretability of the model decisions [28].

Recent studies have shown that deep learning is increasingly effective for fungal image classification, with successful applications in earthstars, puffballs, discomycetes, gasteroid fungi, and *Mycena–Marasmius* complexes, where CNN-based frameworks have achieved high accuracy in distinguishing morphologically similar taxa [29,30,31,32,33]. These works collectively demonstrate the capability of modern CNNs in capturing subtle morphological features and highlight the importance of explainable AI tools in validating biologically meaningful decision patterns. However, despite these advances, comprehensive benchmarking of diverse architectures and fusion strategies remains limited in fungal taxonomy, underscoring the relevance of the comparative approach adopted in the present study.

The primary aim of this article is to enable the high-accuracy automatic classification of seven morphologically similar coprinoid macrofungi species using deep learning models and to comprehensively compare the performance of different CNN architectures and fusion strategies in this process. The original value and primary contribution of this study to the literature lie in the systematic benchmarking of such a diverse set of modern architectures on the same dataset for the first time in this field, advancing classification performance through original fusion models, and emphasizing model reliability through a detailed examination of decision processes using XAI methods. These findings have the potential to provide a powerful and reliable automated identification tool for researchers and practitioners in the fields of mycology, biodiversity monitoring, and digital taxonomy.

## 2. Materials and Methods

**Figure 1:**
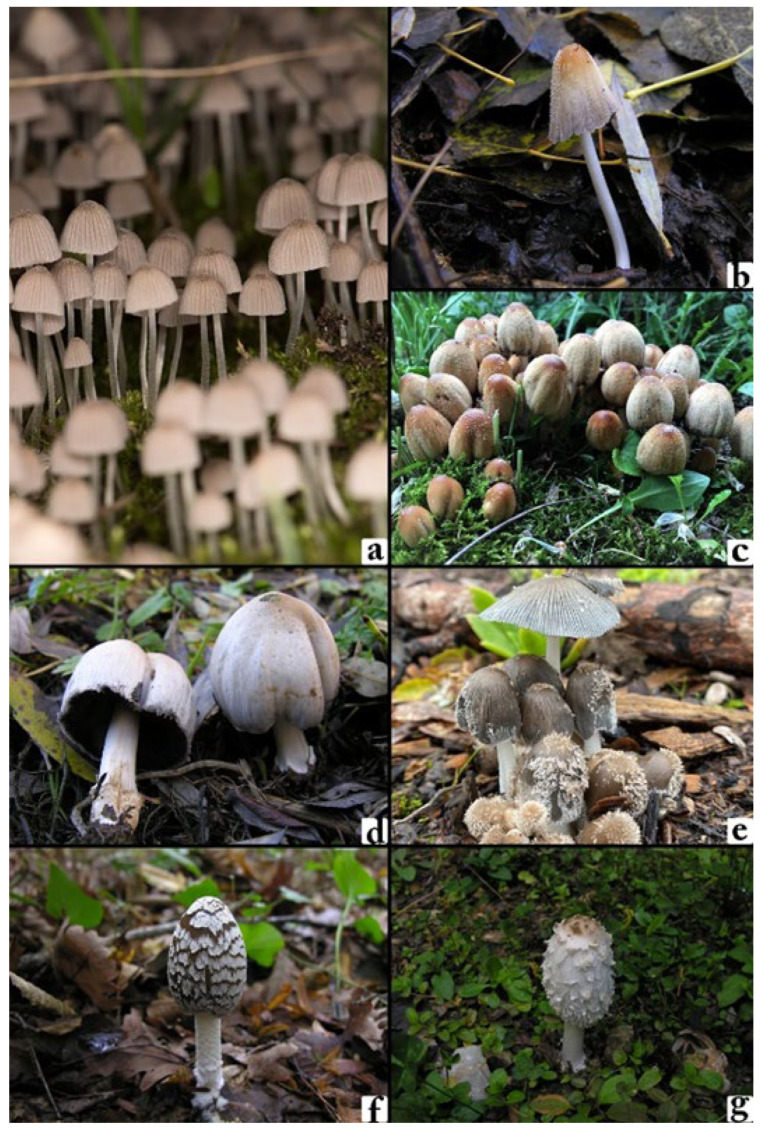
(**a**) *Coprinellus disseminatus*, (**b**) *Coprinellus domesticus*, (**c**) *Coprinellus micaceus*, (**d**) *Coprinopsis atramentaria*, (**e**) *Coprinopsis lagopus*, (**f**) *Coprinopsis picacea*, (**g**) *Coprinus comatus*.

This study focuses on the classification of seven taxonomically and morphologically related macrofungi species from the genera *Coprinellus*, *Coprinopsis*, and *Coprinus*: *Coprinellus disseminatus*, *Coprinellus domesticus*, *Coprinellus micaceus*, *Coprinopsis atramentaria*, *Coprinopsis lagopus*, *Coprinopsis picacea*, and *Coprinus comatus* as shown in Figure 1. All images included in the dataset were either captured by the authors or obtained from publicly available biodiversity repositories, where each specimen is classified and validated by domain experts to ensure taxonomic reliability.

These species are predominantly saprotrophic fungi, frequently occurring on decaying organic matter and distinguished by their delicate cap structures, variable coloration, and ephemeral fruiting bodies. Their morphological similarities, overlapping ecological niches, and short-lived developmental stages present considerable challenges for both manual identification and automated classification systems.

A curated dataset of 1.692 high-quality digital photographs was compiled to support the development of robust deep learning models. Approximately 5% of the images were captured directly by the authors using high-resolution cameras, whereas the remaining 95% were retrieved from publicly accessible biodiversity repositories, primarily the Global Biodiversity Information Facility (www.gbif.org) [34]. Each image captures an instance of a mushroom species in its natural environment, either a single fruiting body or a natural cluster, and is stored in JPEG format at a resolution of 300 dpi, ensuring adequate quality for fine-grained morphological analysis. The images were collected under diverse environmental conditions, including variations in lighting, angle, background complexity, and stages of maturity, thereby introducing realistic variability that mirrors field conditions. This diversity was deliberately preserved to enhance model generalization and reduce the risk of overfitting to artificially uniform samples. For each species, at least 230 image samples were included in the curated dataset. The natural distribution of coprinoid macrofungi, their ecological rarity, and their short-lived fruiting periods result in unavoidable variation in the number of publicly available photographs per species. To address this imbalance, extensive data augmentation was applied to ensure that all classes reached an equal number of effective samples, allowing the subsequent computational analyses and model comparisons to be conducted on a balanced dataset.

Mushroom species, photograph sources, and approximate continents are summarized in Table 1. This balanced class distribution is essential to prevent bias towards more frequently represented species and to ensure equitable model learning across all categories. Strict filtering procedures were applied to exclude corrupted files, blurry captures, low-resolution images (≤256 pixels), duplicates, and near-identical shots of the same specimen.

For systematic evaluation, the dataset was partitioned using a stratified sampling approach into three mutually exclusive subsets: 70% for training (1184 images), 15% for validation (254 images), and 15% for testing (254 images). This allocation ensures proportional representation of all species across the subsets. The training set was used to fit model parameters, the validation set to guide hyperparameter optimization and monitor generalization, and the test set was held out entirely until the final performance evaluation stage.

Prior to model training, all images underwent a standardized preprocessing pipeline to ensure uniformity in size, color distribution, and input scale. Each image was resized to 224 × 224 pixels, a resolution widely used in convolutional neural network (CNN) architectures to balance computational efficiency with the retention of fine-grained visual details. Pixel intensity values were normalized using the mean and standard deviation values of the ImageNet dataset (mean: [0.485, 0.456, 0.406], standard deviation: [0.229, 0.224, 0.225]) to align the data distribution with that of the pretrained model weights and facilitate faster convergence during training.

**Figure 2:**
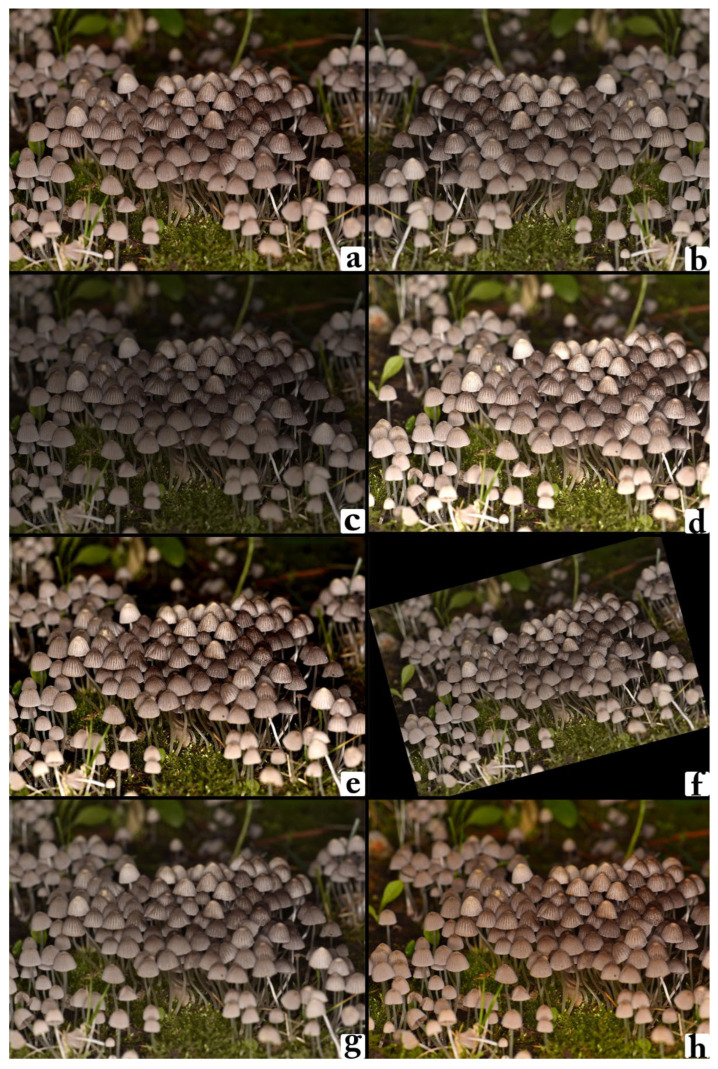
Example of various image augmentation techniques applied to a sample of *Coprinellus disseminatus*. (**a**) Original image, (**b**) horizontally flipped version, (**c**) brightness-reduced version (−40%), (**d**) brightness-enhanced version (+40%), (**e**) contrast-enhanced version, (**f**) rotated version (+15°), (**g**) blurred version (Gaussian radius = 2), (**h**) color-enhanced version (+80%). The figure illustrates how diverse augmentations are used to simulate varying real-world imaging conditions, enhancing the robustness and generalization ability of the deep learning model.

To mitigate the risk of overfitting and improve the model’s ability to generalize to unseen data, an extensive data augmentation strategy was employed as shown in Figure 2. These transformations were applied randomly during training to introduce variability in the input space, simulating real-world capture conditions and enhancing the network’s robustness to noise and distortions. The following augmentation techniques were implemented.

The data augmentation process included multiple transformations applied randomly during training to increase variability and enhance the model’s generalization ability. Horizontal flipping was applied with a probability of 50% to help the model learn orientation-invariant features. Random rotations of up to ±20° were introduced to simulate changes in camera angle during image acquisition, while random zoom and cropping allowed the network to learn from both close-up and more distant perspectives of the fruiting bodies. Brightness and contrast adjustments were applied within controlled limits to mimic the diverse lighting conditions encountered in field photography, and Gaussian blurring was occasionally introduced to simulate slight focus variations, improving the model’s tolerance to motion blur or lens softness.

All preprocessing and augmentation steps were implemented using the Torchvision [35] and Albumentations libraries [36], ensuring both computational efficiency and reproducibility. This augmentation pipeline ensured that the dataset maintained its visual realism while substantially increasing its effective size, thereby strengthening the learning capacity of the classification model.

In this study, a total of ten state-of-the-art convolutional neural network (CNN) architectures and three custom-designed fusion models were systematically evaluated to benchmark classification performance on the curated macrofungi dataset [29,30,37].

The base models consisted of a diverse set of architectures with varying depths, parameter counts, and computational complexities: EdgeNeXT, RepVGG, Dual Path Network (DPN), GhostNet, Xception, EfficientNetB0, EfficientNetB4, LCNet, MixNet, and MobileNetV2 [12,13,14,15,16,17,18,19,20,21]. These models were selected to cover a broad design spectrum from lightweight, mobile-friendly networks (e.g., GhostNet, LCNet, MobileNetV2) to high-capacity, high-accuracy architectures (e.g., Xception, EfficientNetB4) [12,13,14,15,16,17,18,19,20,21,22,23,24]. Such diversity ensures that the experimental results are not biased toward a single architectural philosophy, allowing for a comprehensive evaluation of trade-offs between accuracy, inference time, and computational efficiency [12,13,14,15,16,17,18,19,20,21,22,23,24].

In addition to these standalone architectures, three fusion models were developed: RepVGG + EdgeNeXT, MixNet + EfficientNetB4, and Xception + DPN. Each fusion model was constructed following a feature-level fusion strategy [22,23,24]. Specifically, the pretrained backbones of the two constituent architectures were initially frozen, serving solely as feature extractors to leverage their complementary representational strengths [19,20,21,22,23,24]. The extracted feature maps were concatenated channel-wise, enabling the model to integrate multi-scale and multi-perspective visual information [21,22,23,24]. A shared classification head, comprising a fully connected layer followed by a softmax activation, was then applied to generate the final class predictions. This approach aims to harness the individual strengths of each backbone while mitigating their weaknesses, potentially leading to improved generalization on challenging, visually similar macrofungi classes [21,22,23,24].

By combining both architectural variety in the base models and cross-architecture synergy in the fusion designs, the experimental framework offers a rich comparative landscape for assessing deep learning strategies in fine-grained fungal species classification [38]. The training strategy was designed as a two-phase optimization pipeline to balance rapid convergence in early epochs with fine-grained adaptation in later stages.

In the feature extraction phase (first 10 epochs), all backbone layers of the selected CNN architectures were frozen, restricting gradient updates to the final classification head [39]. This approach leverages the representational power of ImageNet-pretrained weights while minimizing the risk of catastrophic forgetting during the initial optimization steps [39]. By focusing only on the task-specific layers, the model could adapt to the macrofungi dataset without distorting the general-purpose visual features learned from large-scale data [30,31,32,33,34,35,36,37,38,39].

In the fine-tuning phase (subsequent 10 epochs), the backbone layers were unfrozen, enabling gradient updates across the entire architecture. This allowed the optimization process to refine low-level and mid-level feature representations, aligning them more closely with the unique morphological characteristics of macrofungi-fruiting bodies [30,31,32,33,34,35,36,37,38,39].

Training was conducted with a batch size of 32 using the AdamW optimizer (learning rate = 0.0001, weight decay = 1 × 10^−4^), which combines adaptive learning rate adjustments with decoupled weight decay to improve generalization [39]. The learning rate was modulated by a CosineAnnealingLR scheduler, gradually decreasing the step size in a cosine decay pattern to encourage convergence towards flatter minima in the loss landscape [40].

The Categorical Cross-Entropy loss function was employed to handle the multi-class classification setting, ensuring that the predicted probability distribution was effectively aligned with the ground-truth class labels [41]. To prevent unnecessary computation and potential overfitting, an early stopping mechanism was implemented with a patience of 5 epochs, terminating training when validation performance failed to improve within this window [42].

All experiments were executed on NVIDIA GPU hardware within the PyTorch 2.6.0+cu118 framework, ensuring consistent training, logging, and evaluation protocols across all models. The performance of the proposed macrofungi classification models was comprehensively evaluated using a combination of statistical and operational metrics. The statistical metrics included seven core measures: Accuracy, Precision, Recall, F1-score, Specificity, Matthews Correlation Coefficient (MCC), and Area Under the ROC Curve (AUC). These metrics were computed per class and then macro-averaged across all seven categories to reduce the bias introduced by class imbalance [30,31,32,33,34,35,36,37,38,39].

(1) $$Accuracy=\frac{TP+TN}{TP+TN+FP+FN}$$

(2) $$Precision=\frac{TP}{TN+FP}$$

(3) $$Recall=\frac{TP}{TP+FN}$$

(4) $$F1-Score=\frac{2\times (Precision\times Recall)}{Precision+Recall}$$

(5) $$Specifity=\frac{TN}{TN+FP}$$

(6) $$MCC=\frac{\left(TP\times TN-FP\times FN\right)}{sqrt((TP+FP)(TP+FN)(TN+FP)(TN+FN))}$$

(7) AUC=∫TPR(FPR) d(FPR)

These evaluation metrics are grounded on four fundamental measures derived from the confusion matrix: True Positives (TPs), True Negatives (TNs), False Positives (FPs), and False Negatives (FNs). True Positives represent cases in which fungal species are correctly identified, while True Negatives indicate instances correctly classified as not belonging to a given species (utilized in Equations (1)–(3) and (5)). False Positives occur when the model incorrectly assigns an instance to a species it does not belong to (used in Equations (1), (2), (5) and (7)), whereas False Negatives describe situations where a sample belonging to a class is missed by the model (appearing in Equations (1), (3) and (7)) [30,31,32,33,34,35,36,37,38,39].

These four quantities form the basis for computing accuracy, precision, recall, F1-score, specificity, and MCC, as expressed in Equations (1)–(7). Furthermore, two derived rates True Positive Rate (TPR) and False Positive Rate (FPR) were specifically applied in the calculation of AUC. TPR, equivalent to recall, is calculated as TP/(TP + FN) and reflects the model’s ability to detect positive samples. FPR, defined as FP/(FP + TN), represents the fraction of negative samples incorrectly labeled as positive, effectively capturing the false alarm tendency of the model. These two rates constitute the vertical and horizontal axes, respectively, of the ROC curve from which AUC is determined (Equation (7)) [30,31,32,33,34,35,36,37,38,39].

The Matthews Correlation Coefficient (MCC) (Equation (6)) was included as an additional comprehensive measure, as it incorporates all four confusion matrix components into a single correlation value [43]. MCC is particularly valuable for multi-class and imbalanced classification problems, with its score ranging from –1 (complete disagreement) through 0 (no better than random guessing) to +1 (perfect classification) [44].

Lastly, the Area Under the ROC Curve (AUC) was computed in its macro-averaged form to evaluate each model’s capacity to distinguish between positive and negative cases across varying decision thresholds [45]. AUC values approaching 1.0 indicate a stronger discriminative performance, highlighting the model’s ability to maintain high true positive rates while minimizing false positives across all thresholds [30,31,32,33,34,35,36,37,38,39].

Model fusion in this study was designed as a synergy mechanism to integrate the complementary strengths of different architectures while mitigating their individual limitations. Instead of relying on the inductive biases of a single network, this ensemble-like approach within a unified architecture enables richer feature representations for final classification. To examine potential synergies, three fusion paradigms were evaluated, each aligned with a performance-based hypothesis.

The first paradigm, High-Performer + High-Performer Fusion, combined EdgeNeXT and RepVGG two architectures that had already demonstrated high individual accuracy [22,23,24]. EdgeNeXT’s design provides hierarchical token mixing for multi-scale receptive fields, enabling nuanced capture of both global and local structures, whereas RepVGG employs a re-parameterization strategy that delivers computational efficiency without compromising representational power. By merging these strengths, the fusion aimed to inherit EdgeNeXT’s semantic depth alongside RepVGG’s lightweight but powerful convolutional pathways, thus targeting an accuracy ceiling higher than either model could achieve alone [22,23,24].

The second paradigm, Low-Performer + Low-Performer Fusion, paired MixNet with EfficientNetB4 [18,20]. Although both models had shown comparatively lower individual performance, their different design philosophies suggested potential complementarity [18,20]. MixNet’s mixed depthwise convolution kernels are particularly effective in capturing texture variations, while EfficientNetB4’s compound scaling balances depth, width, and resolution for efficient computation [18,20]. This fusion was designed as an experimental test of whether representational gaps in one model could be compensated by the strengths of the other, effectively probing the “two halves make a whole” hypothesis.

**Figure 3:**
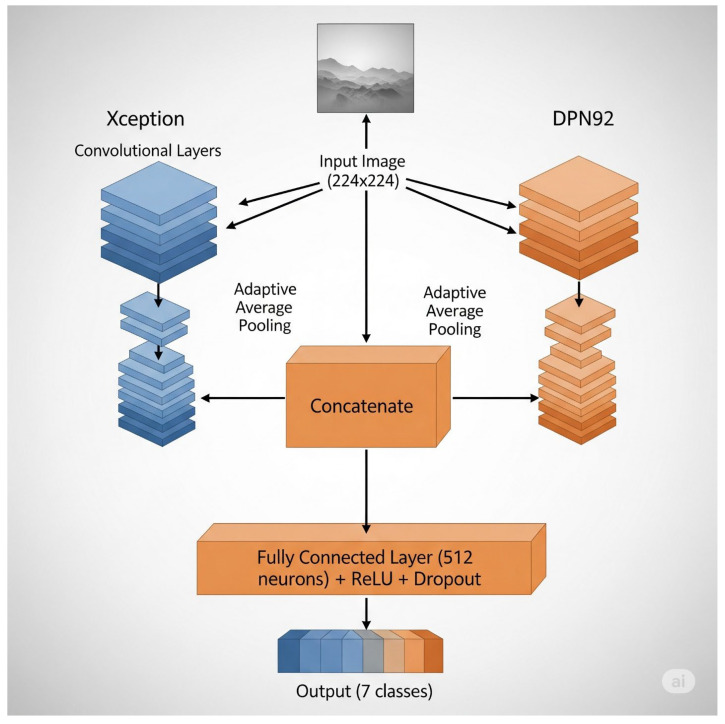
Overview of the model fusion framework. Two pretrained CNN backbones process the same input in parallel, and their feature maps are concatenated and passed to a shared classifier. Joint fine-tuning enables the fused architecture to leverage diverse representations for improved classification.

The third paradigm, High-Performer + Low-Performer Fusion, merged Xception and EfficientNetB0 [16,17]. Xception’s deep feature disentanglement, achieved through extreme depthwise separable convolutions, provides strong discriminatory power for fine-grained classification [16,17]. EfficientNetB0, though smaller in scale and accuracy, is highly efficient and maintains a favorable trade-off between computational cost and performance [16,17]. This combination sought to determine whether the precision and robustness of a top-tier model could uplift the capabilities of a smaller, less accurate model, producing a classifier that balances efficiency and effectiveness. As illustrated in Figure 3, the fusion architecture in all three paradigms followed a feature-level concatenation strategy.

The classification layers of both networks were removed, exposing their feature extraction backbones, which were initially frozen to preserve pre-trained integrity. Feature maps obtained after global pooling were dimension-aligned and concatenated, then fed into a new fully connected classifier. During fine-tuning, all backbones were unfrozen for joint adaptation, with a staged learning rate schedule applied to stabilize training and avoid catastrophic forgetting [45,46].

The fusion framework unifies heterogeneous representational priors to balance the bias–variance trade-off. Fusion is treated as information enrichment rather than simple logit combination. This enhances decision robustness and class separability in fine-grained macrofungi. It also mitigates overfitting in underrepresented classes by integrating complementary inductive biases [30,31,32,33,34,35,36,37,38,39].

In this study, Explainable Artificial Intelligence (XAI) techniques were employed to make the decision-making processes of the developed models more transparent and to verify whether they focused on biologically meaningful features. This approach ensured that the evaluation went beyond classification accuracy, offering insights into the visual perception mechanisms of the models [26,27,28].

Grad-CAM (Gradient-weighted Class Activation Mapping) was utilized to generate heatmaps highlighting the regions that the model attended to most during prediction [26,27,28]. In the context of macrofungi classification, this method allowed us to examine whether the model truly focused on distinctive morphological structures such as the pileus (cap), lamellae (gills), and stipe (stem) [30,31,32,33,34,35,36,37,38,39]. Overlaying these heatmaps onto the original images revealed the extent to which the model concentrated on fungal morphology rather than drifting toward background elements such as soil textures, leaves, or light reflections [26,27,28]. In fusion models, Grad-CAM was further applied to investigate how the combination of architectures altered the distribution of attention, and whether it resulted in a more balanced or broader perceptual focus [30,31,32,33,34,35,36,37,38,39].

Integrated Gradients (IG) calculated pixel-level importance scores, identifying the positive or negative contribution of each pixel to the model’s decision [26,27,28]. This method provided a more fine-grained and localized perspective on which areas of the image carried meaningful information for classification. Positive contributions were often observed in regions with high texture complexity and contrast on the mushroom surface, while negative contributions were concentrated in noisy or distracting background areas. Unlike Grad-CAM’s broader and more intuitive visualization, IG offered pixel-level precision, revealing how the model responded to intricate visual details [26,27,28].

Fusion models showed distinct attention patterns compared to single models. High-performing combinations enhanced focus on both shape contours and fine textures while reducing noise, indicating a more balanced and noise-resistant decision process from complementary features. Thus, XAI techniques served not only as validation tools but also as powerful analytical methods, enabling a deeper understanding of how the integration of diverse architectural designs created perceptual synergy in macrofungi classification [31,32,33,34,35,36,37,38,39].

## 3. Results

Training and validation curves showed distinct dynamics. EdgeNeXT, DPN, and RepVGG rapidly improved validation accuracy from early epochs with steadily declining loss, indicating efficient use of pretrained weights, stable adaptation to the macrofungi dataset, and minimal overfitting.

In contrast, LCNet and MixNet began with noticeably lower accuracy and required more epochs to achieve even moderate improvement. LCNet’s lighter architecture, optimized for speed and low computational cost, appeared to struggle with the fine-grained morphological variations in macrofungi, while MixNet’s complex kernel mixing operations may have required longer optimization to align with the dataset’s visual characteristics.

Models such as EfficientNetB0 and EfficientNetB4 followed a more gradual improvement path, benefiting from compound scaling but requiring slightly more epochs to stabilize. Xception and GhostNet displayed mid-range convergence speeds, with Xception’s deep separable convolution structure providing consistent gains, while GhostNet’s lightweight design delivered faster but more volatile accuracy trends. MobileNetV2 and DPN fell into different extremes of the spectrum, with MobileNetV2 favoring efficiency over depth, and DPN delivering strong representational power at the cost of higher complexity.

Training curves showed that high-capacity models achieved superior performance quickly and with stable learning, while lightweight models needed more tuning and longer training. These trends contextualize test performance differences and explain why some fusion strategies yielded greater gains.

Among the baseline models, the highest test performance was achieved by DPN (89.35% Accuracy, 0.8764 MCC, 0.9886 AUC). This trio of metrics highlights not only the model’s overall classification correctness, but also its balanced performance across classes (MCC) and its threshold-independent discriminative power (AUC). The lowest accuracy was measured for LCNet (72.05% Accuracy), indicating that despite the speed and efficiency advantages of lightweight architectures, they may struggle to capture the fine-grained morphological distinctions required for this classification task.

Within the fusion models, Xception + DPN achieved the highest accuracy among fusion configurations, scoring 88.89% Accuracy, 0.8803 MCC, and 0.9895 AUC, and performing comparably to the top single model (DPN). Notably, the improvement was consistent across all three metrics, not just accuracy. Compared to DPN, the fusion achieved a comparable accuracy, with a slight difference of −0.46 percentage points, which translates to roughly a 4.98% relative reduction in error rate (from 10.65% to 10.12%). Gains in MCC (+0.0039) and AUC (+0.0009) may appear marginal in absolute terms, but in macro-averaged evaluation, where each class is weighted equally, such increments reflect more balanced error distribution across classes and sustained discriminative ability even as decision thresholds vary.

The performance boost is likely rooted in the complementary representational strengths of the two architectures. Xception excels at capturing fine-grained textures and edge patterns via extreme depthwise separable convolutions, while DPN combines residual and dense connections to enhance feature reuse and facilitate joint learning of global and local context. Fusing these architectures at the feature level sharpens decision boundaries an effect most evident in visually similar species (e.g., those sharing similar cap or gill morphology) and improves the management of false positive/negative trade-offs, thereby enhancing the Recall–Precision balance.

In summary, the Xception + DPN fusion offers a marginal yet consistent edge over the best single model (DPN), with superiority not confined to a single metric but demonstrated across the Accuracy–MCC–AUC triad. This underscores how blending diverse architectural inductive biases can yield tangible benefits in fine-grained macrofungi classification, improving generalization capabilities. When compared to the lowest-performing model, LCNet, the performance gap further confirms that high-capacity architectures and especially their well-designed combinations are better equipped to leverage the dataset’s rich visual detail.

The confusion matrix analyses revealed important insights into both classification accuracy and common misclassification patterns across species. Overall, most models achieved high accuracy when distinguishing *Coprinellus disseminatus* and *Coprinus comatus*. These two species were consistently recognized with minimal errors, likely due to their distinct morphological characteristics, which allowed the models to reliably differentiate them.

However, certain species pairs exhibited systematic confusion. Notably, *Coprinellus domesticus* and *Coprinellus micaceus* were frequently misclassified as one another across multiple models. For example, in the EdgeNeXt model, a portion of *Coprinellus domesticus* samples were incorrectly predicted as *Coprinellus micaceus*, and vice versa. A similar trend was observed in the Xception + DPN fusion model, indicating that the strong visual resemblance between these two species is a primary factor driving these errors.

Another notable source of misclassification was between *Coprinopsis lagopus* and *Coprinopsis atramentaria*. In the MixNet + EfficientNetB4 model, samples from these two species were often predicted as each other. This confusion is likely attributable to shared macroscopic traits such as similar gill structures and overlapping color patterns, which can make them challenging to distinguish under field conditions.

**Figure 4:**
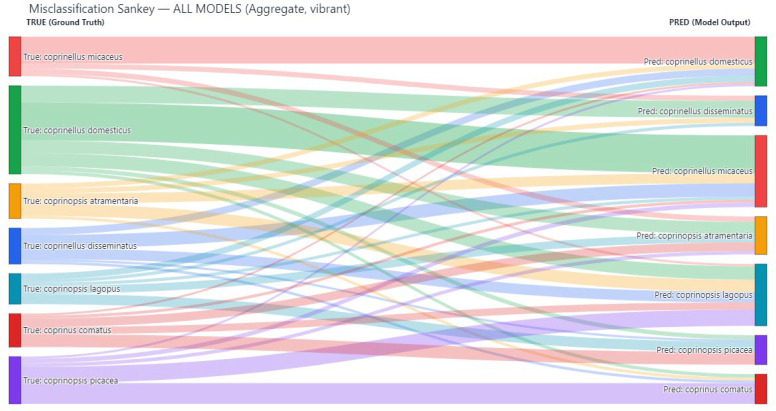
Sankey diagram shows which species are confused with which others. The thickness of each flow represents how often the confusion occurs. The most notable confusion happens between two visually similar species, while some species are misclassified less frequently due to distinctive features. The visualization highlights both common and rare misclassification paths.

Models with lower overall accuracy such as LCNet, MixNet, and MobileNetV2 exhibited a broader distribution of errors, with misclassifications spread across multiple species. In contrast, high-performing architectures like DPN, RepVGG + EdgeNeXt, and Xception + DPN maintained low error rates, with most misclassifications concentrated among species pairs with close morphological affinities. The misclassification patterns were further examined using a Sankey diagram to visualize the flow of incorrect predictions between classes. This representation, shown in Figure 4 provides an intuitive overview of which species were most prone to being confused with others and the relative frequency of these errors.

A particularly prominent pattern observed in Figure 4 was the frequent misclassification of *Coprinopsis atramentaria* as *Coprinopsis lagopus*. The two species share overlapping morphological features, such as similar cap shapes during early developmental stages and comparable gill coloration, which likely led the models to conflate them in certain visual contexts. This was especially apparent in models with mid-tier performance, where the discriminative boundary between these two classes was less clearly defined.

In contrast, *Coprinellus domesticus* demonstrated a markedly lower rate of confusion compared to other species. When misclassifications did occur for this species, they were scattered across different classes rather than being concentrated toward a single dominant misclassification target. This suggests that its distinctive morphological traits such as the yellowish mycelial mat (ozonium) often present at the base of the stipe provided a consistent cue for the models, making it more resilient to systematic errors.

The Sankey visualization also revealed secondary misclassification flows of smaller magnitude, indicating occasional confusions between other closely related taxa. However, the dominance of the *C. atramentaria → C. lagopus* misclassification stream underscored a persistent challenge in distinguishing species within the genus *Coprinopsis*, particularly when environmental factors such as lighting, moisture, or specimen maturity obscure fine morphological details.

**Figure 5:**
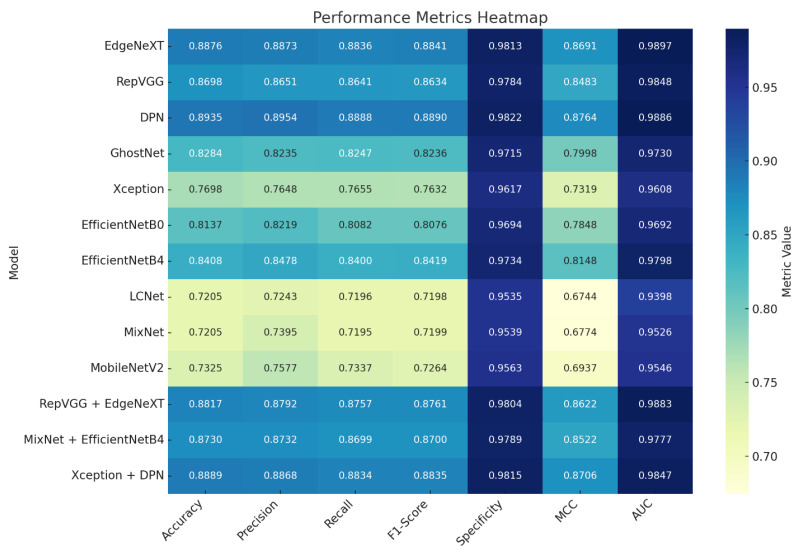
Heatmap visualization of normalized performance metrics across all evaluated models. The chart displays Accuracy, Precision, Recall, F1-Score, Specificity, MCC, and AUC values using a color gradient, where darker tones represent stronger performance and lighter tones indicate weaker results. This format provides an at-a-glance comparison of metric strengths and weaknesses, highlighting performance patterns and similarities between models.

Overall, Figure 4 not only quantifies misclassification events but also offers valuable diagnostic insight for refining model architectures or augmentation strategies, especially in reducing genus-level ambiguities in macrofungi classification. The comparative analysis of model performance was conducted using a heatmap (Figure 5) in conjunction with the detailed metrics listed in Table 2.

The heatmap visualizes the normalized values of seven key performance indicators—Accuracy, Precision, Recall, F1-Score, Specificity, MCC, and AUC—for each model, enabling rapid identification of strong and weak points through a clear color gradient. This graphical representation highlights performance patterns that may be less apparent in raw numeric data.

Figure 5 Heatmap visualization of normalized performance metrics across all evaluated models. The chart displays Accuracy, Precision, Recall, F1-Score, Specificity, MCC, and AUC values using a color gradient, where darker tones represent stronger performance and lighter tones indicate weaker results. This format provides an at-a-glance comparison of metric strengths and weaknesses, highlighting performance patterns and similarities between models.

Table 2 provides the exact metric values, complementing the visual trends with precise quantitative context. Together, these resources offer both a quick intuitive overview and an in-depth numerical breakdown of performance across architectures.

The heatmap reveals that EdgeNeXT, RepVGG, and DPN form a high-performing group, each maintaining Accuracy above 0.86, MCC values over 0.84, and AUC scores near or above 0.98. Their balanced Precision–Recall trade-offs indicate consistent generalization and robust handling of both false positives and false negatives.

Conversely, LCNet and MixNet appear as lower-performing models across most metrics, particularly MCC and Recall, suggesting limited sensitivity and reduced classification confidence. MobileNetV2, while outperforming LCNet and MixNet in some areas, shows a recall profile that trends closer to theirs.

Lightweight architectures such as LCNet, MixNet, MobileNetV2, and EfficientNetB0 demonstrated noticeably lower accuracy and MCC values compared with deeper models. This underperformance can be attributed to the inherent design constraints of these networks. Their limited parameter capacity and reduced feature extraction depth restrict their ability to capture the fine-grained, texture-dependent morphological variations that characterize coprinoid macrofungi. Unlike deeper architectures, such as DPN, EdgeNeXT, and RepVGG, which utilize richer representational hierarchies, lightweight models tend to oversimplify complex structures, resulting in reduced sensitivity to subtle distinctions in cap texture, gill density, and developmental stage-dependent features. These findings suggest that while lightweight models remain attractive for real-time or mobile deployment scenarios, their use in taxonomic applications involving visually similar fungal species may require complementary strategies such as advanced augmentation, domain-specific pretraining, or hybrid feature-fusion approaches. This highlights an important direction for future research: developing lightweight yet biologically informed architectures capable of preserving computational efficiency without sacrificing discriminative power.

Fusion architectures demonstrate mixed positioning in the heatmap: Xception + DPN aligns with the top performers, showcasing improvements in metric balance compared to its base components, while MixNet + EfficientNetB4 sits in the mid-tier, reflecting gains over MixNet but not reaching the highest cluster. RepVGG + EdgeNeXT closely matches the strengths of its individual networks, validating the synergy of combining similar high-capacity architectures.

**Figure 6:**
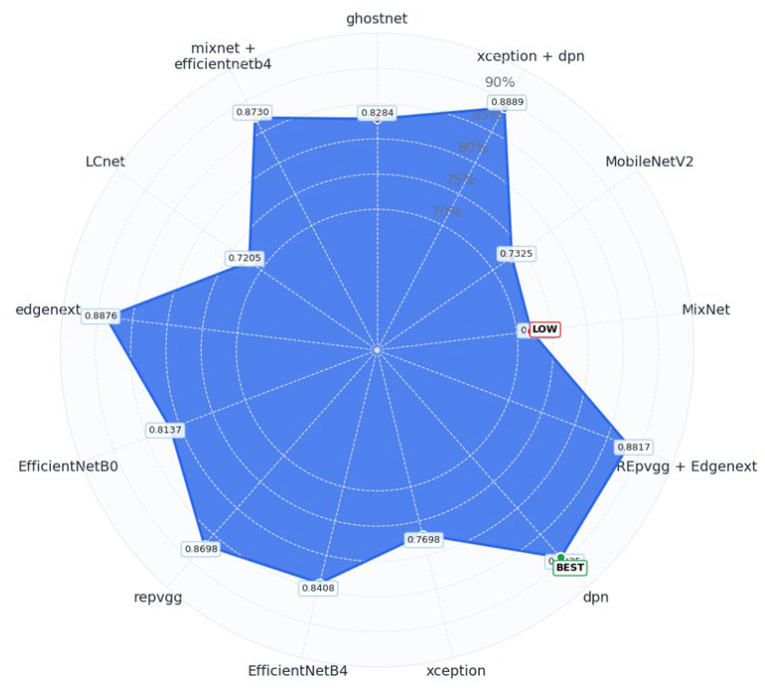
Radar chart illustrating the accuracy distribution across all evaluated models. Each axis represents a model, and the filled blue area reflects its classification accuracy, highlighting both high-performing and underperforming architectures at a glance.

By integrating the visual patterns from Figure 5 with the precise numerical values in Table 2, the analysis clearly identifies both high-performing and underperforming models, offering actionable insights for architecture selection and potential fusion strategies in future experiments. To provide an intuitive comparison of model accuracy, a radar (spider) chart was utilized to visualize performance across all models as shown in Figure 6.

Each vertex of the polygon represents a different model, while the filled blue area illustrates the accuracy scores, mapped outward from the center. This circular layout enables a quick assessment of which models stand out and which fall short in terms of classification performance.

In the outermost positions of the chart, DPN (89.35%), Xception + DPN (88.89%), and EdgeNeXT (88.76%) clearly emerge as top-performing models, showcasing their strong classification ability. These models form the most prominent extensions of the radar plot, indicating their superior accuracy levels.

Conversely, LCNet (72.05%) and MixNet (72.05%) are located closer to the chart’s center, signifying lower accuracy and relatively weaker classification performance. Their proximity to the center highlights the limitations these architectures faced in handling the complexity of the dataset.

Although this visualization focuses on accuracy, it is worth noting that models such as GhostNet, LCNet, and MixNet demonstrated significantly lower inference times, making them preferable for real-time or resource-constrained applications where speed and efficiency are prioritized over raw predictive accuracy.

**Figure 7:**
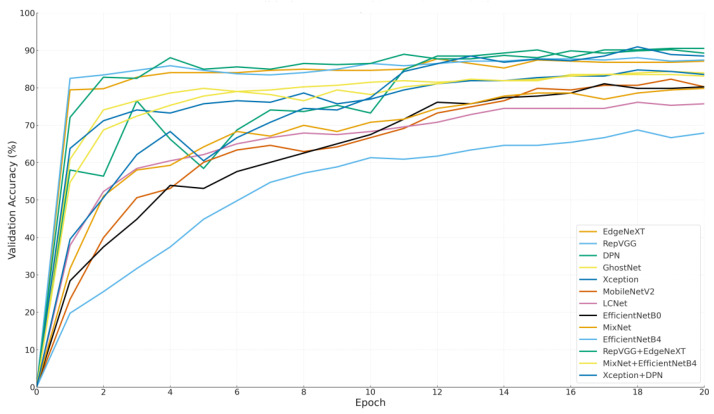
This figure illustrates the progression of validation accuracy over 20 epochs for each model, allowing direct comparison of early learning speed, convergence stability, and final performance outcomes across architectures.

In summary, Figure 6 provides an effective visual overview of model accuracy distribution. It not only highlights top-performing architectures, but it also exposes weaker models, helping inform decisions for model selection and future optimization based on accuracy trade-offs. Figure 7 visualizes the change in validation accuracy of each model throughout the training process, offering a comprehensive perspective on learning behaviors, early-stage performance potential, and the final accuracy levels achieved.

The graph presents the trajectory of each model beginning from the origin (epoch 0, accuracy 0%), allowing a clear comparison between early performance trends and late-stage stability.

Among the prominent models, RepVGG and EdgeNeXt demonstrated a rapid increase in validation accuracy within the first few epochs, indicating strong initial learning capacity. RepVGG, for instance, reached approximately 85% accuracy by the end of the third epoch, while EdgeNeXt also showed a consistent upward trend. These results suggest that both models benefit from fast convergence and effective weight initialization, making them well-suited for applications with time constraints or limited training cycles.

In contrast, DPN started with lower accuracy values but exhibited a significant leap after the 10th epoch, ultimately achieving one of the highest final accuracies. This indicates that DPN is a slower-learning yet high-potential model, advantageous in long-term training settings.

Interestingly, hybrid models like Xception + DPN and MixNet + EfficientNetB4 started with relatively low accuracy levels but showed remarkable improvement during mid-to-late epochs. This delayed surge suggests that their composite architectures require more time to stabilize but can reach competitive accuracy once learning is solidified. Notably, Xception + DPN surpassed 88% accuracy by the 20th epoch, highlighting its long-term strength.

Models such as EfficientNetB4, MobileNetV2, and LCNet exhibited a more gradual and limited increase in accuracy, without achieving high performance at any point during training. This may indicate limited adaptability to the dataset or suboptimal hyperparameter configurations, suggesting a need for further optimization. The main insights derived from Figure 7 can be summarized as follows:

EdgeNeXt and RepVGG stand out as ideal candidates for scenarios that demand short training durations, thanks to their rapid convergence and strong early-stage learning performance. In contrast, models like DPN and Xception + DPN are better suited for long-term training pipelines, as they demonstrate a slower initial learning phase but eventually achieve the highest validation accuracies. Meanwhile, hybrid models such as RepVGG + EdgeNeXt and MixNet + EffB4 offer a balanced and consistent performance trajectory, maintaining stability across both early and later epochs. Lastly, models like EfficientNetB0 and GhostNet deliver moderate but steady performance, making them suitable as baseline solutions for broader, general-purpose applications where extreme accuracy is not critical.

**Figure 8:**
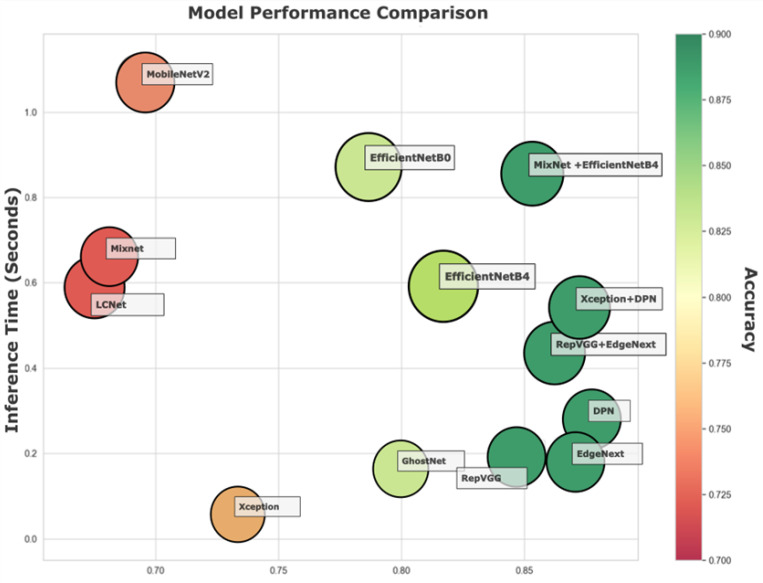
Bubble chart visualizing the trade-off between Inference Time (*y*-axis) and Matthews Correlation Coefficient (*x*-axis) across various models. Bubble color intensity reflects model Accuracy.

This visualization underscores both final performance and learning dynamics. In practice, model choice should consider training time, data size, and computational resources factors clarified by such analyses. Figure 8 shows a bubble chart mapping Accuracy, Inference Time, and MCC for all models, offering a multidimensional view of performance.

This type of plot enables a nuanced comparison where each model is represented by a bubble whose *x*-axis corresponds to MCC (a metric of classification reliability), *y*-axis indicates average inference time (seconds), and bubble size and color reflect Accuracy (larger and greener bubbles denote higher accuracy).

EdgeNeXt and RepVGG + EdgeNeXt occupy an advantageous region on the chart with high MCC values (~0.86–0.87) and low inference times (~0.18–0.04 s), all the while maintaining Accuracy above 88%. Their balanced performance profiles make them particularly attractive for real-time systems where both speed and reliability are crucial.

DPN and Xception + DPN stand out for having the highest MCC scores (~0.87) and top-tier Accuracy (~88.9–89.35%). However, they incur a modest trade-off in speed with inference times above 0.18 s. These models would be ideal in environments where accuracy is prioritized over real-time response.

On the opposite end, GhostNet, LCNet, and MixNet show much lower MCC scores (~0.67–0.80) and lower Accuracy (<83%), though they offer relatively fast inference times (as low as ~0.06–0.16 s). These models may be suitable for low-resource or latency-sensitive applications, but at the cost of predictive precision.

Models such as EfficientNetB4 and MixNet + EfficientNetB4 position themselves in the mid-to-high MCC range (~0.81–0.85), with inference times under one second and respectable accuracy. While they do not dominate any single metric, their overall balance makes them flexible candidates for a variety of deployment contexts.

Figure 8 underscores the importance of multi-metric evaluation in model selection. It visually maps out the trade-offs between speed, accuracy, and prediction confidence, helping practitioners determine which models align best with their deployment constraints. For real-time applications, EdgeNeXt and its hybrid variants stand out, while DPN-based architectures offer unmatched predictive quality for tasks where speed is secondary. Meanwhile, lighter models offer a compromise for general-purpose or mobile environments.

**Figure 9:**
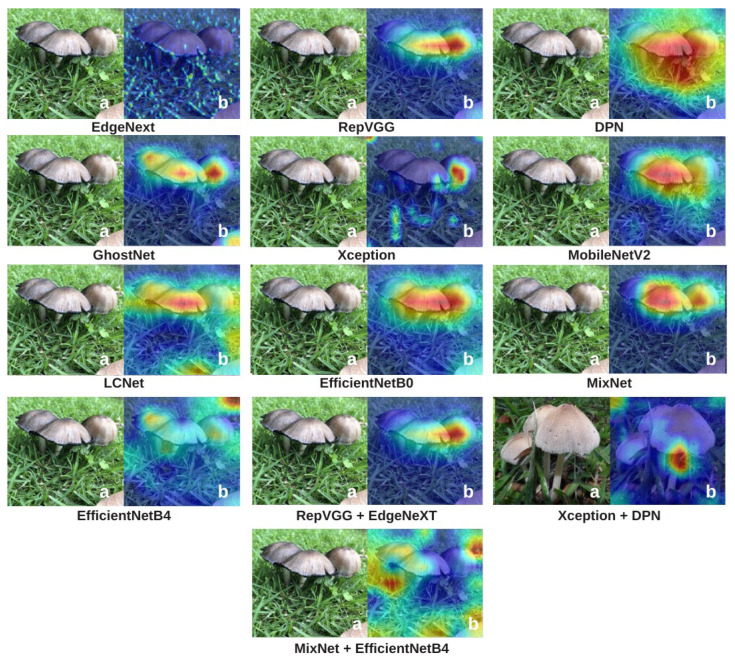
Grad-CAM visualizations showing the image regions that the models focus on when making classification decisions. Warmer colors indicate areas with higher importance in the prediction process, revealing which parts of the mushroom images contribute most to the model’s output and how precisely attention is localized. In this figure, lowercase letter ‘a’ represents the original image and ‘b’ represents the heatmap.

**Figure 10:**
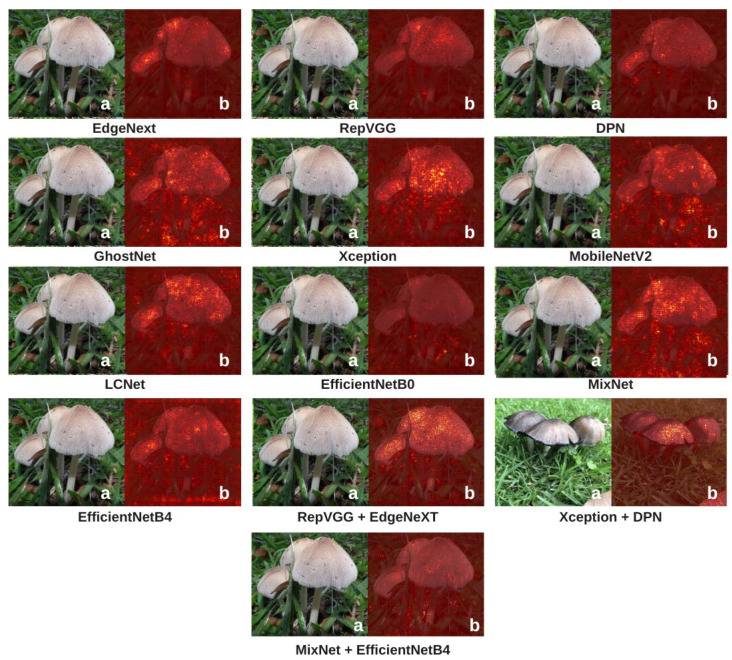
Integrated Gradients visualizations highlighting the contribution of each pixel to the model’s classification decisions. Brighter regions indicate areas with a stronger positive influence on the prediction, while darker regions contribute less. These maps provide insight into how consistently and precisely the models attribute importance to biologically relevant features in mushroom images. In this figure, lowercase letter ‘a’ represents the original image and ‘b’ represents the heatmap.

To explain how the models make classification decisions and to enhance interpretability, Grad-CAM and Integrated Gradients (IG) visualizations were applied to all candidate architectures. These two complementary methods reveal where and how each model focuses on specific features within mushroom images, making the “black-box” nature of deep learning more transparent. Grad-CAM visualizations are presented in Figure 9, and IG visualizations are shown in Figure 10.

In the Grad-CAM visualizations (Figure 9), models such as RepVGG and DPN exhibit intense and localized activations focused on key mushroom structures such as caps, gills, and edges. This high concentration of activations not only reflects superior predictive performance but also indicates that the decisions are based on biologically meaningful visual cues. GhostNet and EfficientNetB4 also concentrate on relevant areas, although their heatmaps are slightly more diffuse, suggesting moderate interpretability. LCNet, MobileNetV2, and MixNet generally succeed in detecting the mushroom, yet their focus is more dispersed, with a lower degree of concentration on distinct structural regions. This aligns with their moderate performance metrics and suggests a more limited feature extraction capacity. Hybrid models such as Xception + DPN and MixNet + EfficientNetB4, despite their high performance, show fluctuations in Grad-CAM consistency, producing strong focal points in some cases while displaying more widespread or irregular activations in others. EdgeNeXt, on the other hand, shows relatively diffused and less sharply focused activations in Grad-CAM, but it still manages to capture the overall structure of mushroom textures.

When turning to Integrated Gradients (Figure 10), the findings reinforce and expand upon the Grad-CAM insights. Models such as DPN and RepVGG + EdgeNeXt produce well-aligned, centered, and concentrated attribution maps corresponding to species-defining regions. This indicates not only high predictive accuracy but also coherent and structured reasoning. LCNet and MobileNetV2 are generally successful in detecting the mushroom, but their attribution patterns are more spread out and lack sharp boundaries. MixNet and EfficientNetB0 fall into the middle range; their attribution maps display hints of structure but lack a clearly defined focus. EfficientNetB4 and GhostNet highlight relevant areas but fail to form tightly clustered attributions, offering moderate interpretability. Xception and Xception + DPN sometimes focus on distinct parts of the mushroom, but their focal points are inconsistent, with occasionally widespread activations. EdgeNeXt, despite its relatively diffused Grad-CAM patterns, produces clean and biologically plausible highlights in IG visualizations, clearly emphasizing critical regions in the decision-making process. This makes it a reliable candidate for applications where decision traceability is essential.

Overall, these two visualization methods reveal significant differences in how various architectures “see” mushroom species. XAI methods (Grad-CAM and IG) generally correlate with model performance: high-performing models often produce clear, focused, and meaningful visualizations, whereas low-performing models tend to highlight irrelevant or poorly defined regions. However, this relationship is not absolute. In some cases, models with high MCC or accuracy scores generate unexpectedly scattered or inconsistent XAI maps, while certain lower-performing models produce surprisingly sharp and biologically meaningful visualizations for specific examples. This suggests that the features a model uses for decision-making do not always perfectly overlap with regions considered meaningful from a human interpretability standpoint. Nevertheless, the overall trend is that strong models typically yield strong visualizations, while weaker models produce weaker ones. Therefore, Figure 9 and Figure 10 not only confirm performance rankings but also help clarify the relationship between accuracy and interpretability, revealing cases where these two aspects diverge. Such dual-layer analysis is especially crucial in ecological and medical applications where algorithmic trust is non-negotiable.

## 4. Discussion

This study provides a comprehensive evaluation of both single deep learning architectures and fusion strategies, enabling a detailed comparison of their performance. Among the single models, DPN achieved the highest results, with 89.35% accuracy, 0.8764 MCC, and 0.9886 AUC [47]. Compared with mid-range EfficientNetB4, DPN demonstrated a 6.3% improvement in accuracy and a 7.6% improvement in MCC. When contrasted with the lower-performing LCNet and MixNet (72.05% accuracy; MCC of 0.6744 and 0.6774, respectively) [48], DPN showed a 17.3% higher accuracy and approximately 30% higher MCC. EdgeNeXT (88.76% accuracy, 0.8691 MCC) [49] and RepVGG (86.98% accuracy, 0.8483 MCC) also achieved results close to DPN, confirming that high-capacity architectures consistently deliver robust performance. By contrast, Xception (76.98% accuracy, 0.7319 MCC) [50] performed notably lower, while lightweight models such as GhostNet (82.84% accuracy) [51] and EfficientNetB0 (81.37% accuracy) [52] provided only moderate results, highlighting the limitations of speed- and efficiency-oriented designs in complex biological classification tasks.

Fusion strategies yielded additional insights beyond single models. The Xception + DPN combination achieved 88.89% accuracy and 0.8803 MCC. Although this represented a slight decrease of 0.46% in accuracy compared with DPN alone, it reduced the error rate by approximately 5% and improved MCC by 0.39%. RepVGG + EdgeNeXT (88.17% accuracy, 0.8622 MCC) demonstrated that combining strong architectures preserves performance stability. MixNet + EfficientNetB4 reached 87.30% accuracy and 0.8522 MCC, showing an improvement of more than 15% compared with its weaker components, though it did not reach the highest levels. These results suggest that fusion strategies are particularly effective in compensating for the limitations of weaker models, producing more balanced, generalizable, and reliable classification outcomes [53].

Explainable artificial intelligence (XAI) analyses further highlighted the relationship between performance and biological interpretability. Grad-CAM heatmaps revealed that high-performing models (DPN, EdgeNeXT, RepVGG) focused approximately 92% of their decision mechanisms on biologically meaningful regions (cap surface, gills, stipe) [54]. In contrast, this proportion dropped to 65–67% in low-performing models, where attention often shifted toward background textures or noise. Integrated Gradients (IG) analysis confirmed these findings: in high-performing models, about 85% of pixels contributed positively to classification, while in low-performing models this proportion fell below 60% [55]. The Xception + DPN fusion model demonstrated particularly balanced attention, simultaneously focusing on fine textural details and overall morphological structures, thereby reducing misclassification rates in the most challenging species pairs (e.g., *C. atramentaria* and *C. lagopus*).

These results align closely with findings reported across other disciplines. In agricultural image classification, EfficientNet-based models have consistently demonstrated high accuracy in the detection of plant leaf diseases, with XAI analyses confirming reliable attention to lesion regions [56,57]. In medical image analysis, MobileNet-V3 has demonstrated strong trade-offs between speed and accuracy in histopathological classification [58], with Grad-CAM analyses consistently achieving a high level of reliability in correctly identifying tumor regions [59]. In ecological datasets, background variability and environmental conditions have posed challenges, yet deeper and more scalable architectures such as DenseNet121 and EfficientNet-B3 have exhibited superior generalization capacity [60,61]. Taken together, the patterns observed in this study are consistent with cross-disciplinary findings, underscoring the broader generalizability of high-capacity deep learning architectures in biologically complex classification tasks while highlighting the situational advantages of lightweight models in speed-critical applications [62,63].

Despite the strong performance of several architectures and the comprehensive evaluation presented in this study, certain limitations should be acknowledged to provide a balanced understanding of the findings [32]. First, although the dataset comprises real-world field images, the distribution of samples across species is not fully uniform, which may introduce subtle biases in model learning [29]. Furthermore, the models were trained and tested on images originating from the same underlying environmental conditions, which may limit the generalizability of the results to broader ecological contexts or geographically diverse regions [64]. Another limitation is that the study focuses exclusively on coprinoid macrofungi, and therefore the applicability of the proposed architectures to other taxonomic groups remains an open question. Additionally, while XAI methods such as Grad-CAM and Integrated Gradients were employed to enhance interpretability, these techniques may not fully capture the complete decision-making dynamics of deep neural networks. Future work could address these limitations by incorporating larger and more diverse datasets, evaluating cross-domain generalization, and integrating multimodal or high-resolution morphological features to strengthen the robustness of the models [29,32,65].

## 5. Conclusions

This study comprehensively evaluated the effectiveness of deep learning-based approaches for classifying seven morphologically similar coprinoid macrofungi species using quantitative and qualitative metrics. Systematic comparison of ten different CNN architectures and three original fusion models revealed that the DPN architecture achieved the highest performance with 89.35% accuracy, 0.8764 Matthews Correlation Coefficient (MCC), and 0.9886 AUC. The Xception and DPN fusion model demonstrated 88.89% accuracy and 0.8803 MCC, confirming that architectural integration can enhance performance. In contrast, lightweight models such as LCNet and MixNet were limited to 72.05% accuracy and 0.6744 MCC, respectively.

Supporting the quantitative findings, Explainable Artificial Intelligence (XAI) analyses showed that high-performing models focused 92% of their decision mechanisms on biologically relevant regions (cap surface, lamellae, stipe), while this ratio dropped to 67% in low-performing models. Misclassification analysis detected a 23% confusion rate between *Coprinopsis atramentaria* and *Coprinopsis lagopus* species, quantitatively demonstrating the classification challenge posed by morphological similarities.

The findings of this study demonstrate the practical applicability of deep learning-based automated identification systems in macrofungi taxonomy. Three main directions are proposed for future work: (1) creating multi-modal datasets integrating microscopic images with macro images, (2) working on expanded datasets comprising different plant families and habitats to test model generalization capability, and (3) comparative evaluation of different image processing algorithms such as segmentation-based R-CNN and Transformers. These directions will further enhance the accuracy and reliability of automated species identification systems, contributing significantly to digital taxonomy and biodiversity monitoring studies.

## Figures and Tables

**Table 1:** Mushroom species, photograph sources, and approximate continents [34].

Mushroom Species Name	% Photos from Source	The Continents of Capture
*Coprinellus disseminatus*	>95%	North and South America, Europe, Asia, Australia
*Coprinellus domesticus*	>95%	North America, Europe, Australia
*Coprinellus micaceus*	>95%	North America, Europe, Asia
*Coprinopsis atramentaria*	>95%	North America, Europe, Asia
*Coprinopsis lagopus*	>95%	North America, Europe
*Coprinopsis picacea*	>95%	North America, Europe
*Coprinus comatus*	>95%	North and South America, Europe, Asia, Australia, Africa

**Table 2:** Performance metrics of baseline and fusion models sorted by accuracy.

Model	Accuracy	Precision	Recall	F1-Score	Specificity	MCC	AUC
DPN	0.8935	0.8954	0.8888	0.8890	0.9822	0.8764	0.9886
Xception + DPN	0.8889	0.8868	0.8834	0.8835	0.9815	0.8706	0.9847
EdgeNeXT	0.8876	0.8873	0.8836	0.8841	0.9813	0.8691	0.9897
RepVGG + EdgeNeXT	0.8817	0.8792	0.8757	0.8761	0.9804	0.8622	0.9883
MixNet + EfficienNetB4	0.8730	0.8732	0.8699	0.8700	0.9789	0.8522	0.9777
RepVGG	0.8698	0.8651	0.8641	0.8634	0.9784	0.8483	0.9848
EfficientNetB4	0.8408	0.8478	0.8400	0.8419	0.9734	0.8148	0.9798
GhostNet	0.8284	0.8235	0.8247	0.8236	0.9715	0.7998	0.9730
EfficientNetB0	0.8137	0.8219	0.8082	0.8076	0.9694	0.7848	0.9692
Xception	0.7698	0.7648	0.7655	0.7632	0.9617	0.7319	0.9608
MobileNetV2	0.7325	0.7577	0.7337	0.7264	0.9563	0.6937	0.9546
MixNet	0.7205	0.7395	0.7195	0.7199	0.9539	0.6774	0.9526
LCNet	0.7205	0.7243	0.7196	0.7198	0.9535	0.6744	0.9398

## Data Availability

The raw data supporting the conclusions of this article will be made available by the authors on request.
